# 2347. Factors Associated with COVID-19 and Influenza Vaccine Coadministration in Rhode Island

**DOI:** 10.1093/ofid/ofad500.1969

**Published:** 2023-11-27

**Authors:** Lisa M Gargano, Dora M Dumont

**Affiliations:** Rhode Island Department of Health, Providence, Rhode Island; Rhode Island Department of Health, Providence, Rhode Island

## Abstract

**Background:**

Co-administration of COVID-19 and influenza vaccines has several advantages, has been advocated by various public health authorities, and should be seen as an opportunity to increase the uptake of both vaccines. Co-administration of vaccines has a substantial role to play in decreasing the number of consultations or provider visits and hence missed opportunities and can increase timeliness of vaccination. The objective of this analysis was to examine factors associated with COVID-19 and influenza vaccine co-administration in Rhode Island (RI).

**Methods:**

Data were from the RI Child and Adult Immunization Registry (RICAIR), which maintains records of all vaccinations administered in the state. Dates of vaccination were from September 1, 2022-March 30, 2023, corresponding with the availability of influenza vaccine in RI. Co-administration was defined as receipt of COVID-19 and influenza vaccine on the same day. Logistic regression was used to examine the association between selected factors and location of co-administration.

**Results:**

There were 473,872 individuals who received either a COVID-19 and/or influenza vaccine during the study period. Of those, 205,486 received both vaccines, of whom 95,599 received both vaccines on the same day. Adults 19-64 years were less likely to receive both vaccines compared to adults 65+ years (42.8% vs. 59.0%), but of those who did, a greater proportion (52.1% vs. 39.8%; p-value < 0.0001) received them on the same day. Compared to other race/ethnicity groups, Hispanic/Latinos were the least likely to get both vaccines (24.9%), but they were the most likely to get them co-administered (53.4%). Of those who received both COVID-19 and influenza vaccines in the same day, 70.0% were vaccinated at a pharmacy, compared to 22.2% at a primary care provider (PCP). Non-Hispanic Blacks and Hispanic/Latinos had higher odds of receiving same day vaccines from their PCP versus a pharmacy than non-Hispanic Whites (AOR 2.7 [95% CI 2.5-2.9] and 5.0 [95% CI: 4.7-5.3], respectively).

**Figure:**
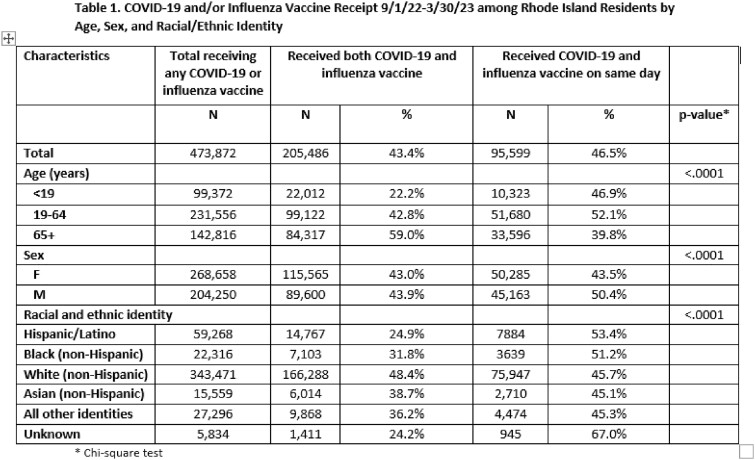

**Figure:**
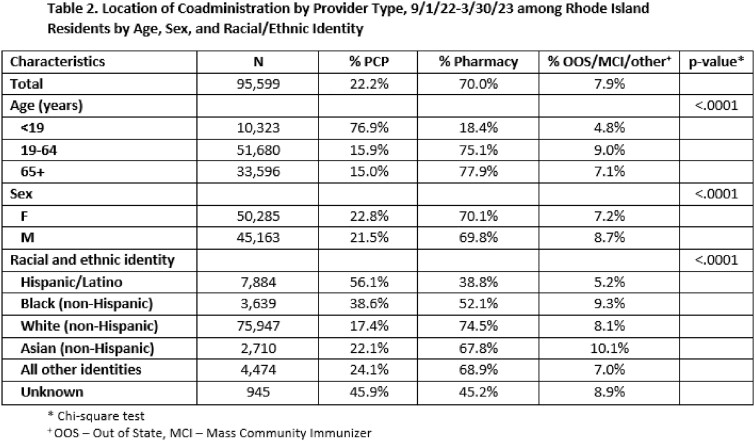

**Figure:**
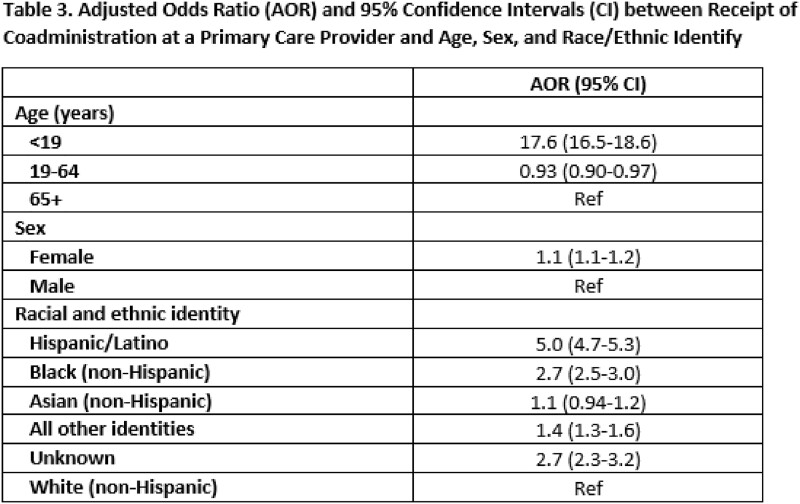

**Conclusion:**

These data demonstrate a willingness to co-administer vaccines among adults. Identifying groups that are less likely to co-administer vaccines and understanding behaviors around where people are getting co-administered will aid in outreach and education efforts.

**Disclosures:**

**All Authors**: No reported disclosures

